# Strong and Localized Luminescence from Interface Bubbles Between Stacked hBN Multilayers

**DOI:** 10.1038/s41467-022-32708-z

**Published:** 2022-08-25

**Authors:** Hae Yeon Lee, Soumya Sarkar, Kate Reidy, Abinash Kumar, Julian Klein, Kenji Watanabe, Takashi Taniguchi, James M. LeBeau, Frances M. Ross, Silvija Gradečak

**Affiliations:** 1 Department of Materials Science and Engineering, Massachusetts Institute of Technology, 77 Massachusetts Ave, Cambridge, MA 02141 USA; 2 Department of Materials Science and Engineering, National University of Singapore, 9 Engineering Drive 1, 117575 Singapore, Singapore; 3 Research Center for Functional Materials, National Institute for Materials Science, 1-1 Namiki, Tsukuba, 305-0044 Japan; 4 International Center for Materials Nanoarchitectonics, National Institute for Materials Science, 1-1 Namiki, Tsukuba, 305-0044 Japan

**Keywords:** Two-dimensional materials, Materials for optics

## Abstract

Extraordinary optoelectronic properties of van der Waals (vdW) heterostructures can be tuned via strain caused by mechanical deformation. Here, we demonstrate strong and localized luminescence in the ultraviolet region from interface bubbles between stacked multilayers of hexagonal boron nitride (hBN). Compared to bubbles in stacked monolayers, bubbles formed by stacking vdW multilayers show distinct mechanical behavior. We use this behavior to elucidate radius- and thickness-dependent bubble geometry and the resulting strain across the bubble, from which we establish the thickness-dependent bending rigidity of hBN multilayers. We then utilize the polymeric material confined within the bubbles to modify the bubble geometry under electron beam irradiation, resulting in strong luminescence and formation of optical standing waves. Our results open a route to design and modulate microscopic-scale optical cavities via strain engineering in vdW materials, which we suggest will be relevant to both fundamental mechanical studies and optoelectronic applications.

## Abstract (web summary)

Optoelectronic properties of van der Waals heterostructures can be tuned via strain. Here, authors demonstrate strong and localized luminescence in the ultraviolet region from interface bubbles between stacked multilayers of hexagonal boron nitride.

## Introduction

Van der Waals (vdW) layered materials and their heterostructures exhibit extraordinary physical properties while readily allowing out-of-plane deformation, which is of great interest for flexible and conformal electronics^1,2^. A unique approach to understand the role of mechanical deformation on optoelectronic properties of vdW materials is to take advantage of the bubbles that are formed during the fabrication of vertical vdW heterostructures. During the stacking process, vdW forces squeeze out and trap a material, such as hydrocarbons^3–5^, air^6^, or water^7,8^, adsorbed on the surface resulting in formation of bubbles at the interface between the stacked layers. Potential applications of bubbles in vdW heterostructures have been recently reported due to their unique structure, the resulting strain, and the pressure in the bubble (vdW pressure)^9,10^. For example, the ability to trap materials under extremely high pressure inside bubbles leads to unusual phenomena such as nano-confined hydrophobic ice^7^ or chemical reactions that would not occur under ambient conditions^11^. Furthermore, bubbles between non-permeable graphene membranes have been used to trap material for liquid cell electron microscopy applications^12^. For optoelectronics and photonics, bubbles in monolayer transition metal dichalcogenides (TMDs) can serve as strain-induced local emitters^13^ and optical cavities^14^. However, the use of bubbles and induced strain to modify optical properties has so far been limited to monolayers. Strain engineering of vdW multilayers should present new opportunities in optoelectronics since multilayers of vdW materials exhibit robust optical performance^15–17^, their bending is different from that seen in a monolayer or in classical plate theory^18^, and multilayers circumvent issues related to exfoliation and manipulation of monolayers.

Here, we study the mechanical behavior of bubbles between stacked multilayers of hexagonal boron nitride (hBN) that we find show a strong localized optical emission. We focus on hBN as a promising material for optoelectronic and quantum optics applications because it exhibits rich optical properties in the ultraviolet region at room temperature^15,19^ with a large band gap of approximately 6 eV^20^. Importantly for applications, hBN emits strong near-band edge emission with large excitonic binding energy^19,21^; its intensity increases with hBN thickness, in contrast to TMDs that are luminescent only in the monolayer limit^22^. The optical emission of hBN can be further enhanced and systematically tuned by the twist angle between two stacked multilayers as a result of moiré superlattice effects^15^. Moreover, single photon emitters attributed to structural defects have been observed in hBN multilayers in a wide spectral range^23,24^.

By combining experimental measurements and theoretical modeling, we show that bubbles formed by bending of vdW multilayers exhibit radius- and thickness-dependent geometry and strain across the bubble, unlike the constant values seen in the case of monolayers. These results provide insights into the bubble geometry as well as the thickness-dependent bending rigidity of vdW multilayers, an important parameter for strain engineering. We observe a strong phonon-coupled luminescence from the hBN bubble regions and discuss its origin and spatial variation. Finally, we demonstrate an approach to tune the bubble geometry and optical properties by utilizing decomposition of the trapped material via electron beam irradiation. This strategy to design effective optical emitters in the ultraviolet region and cavities with controlled dimensions is promising for biological, medical, and stretchable electronic and photonic applications.

## Results

### Interface bubble formation

**Fig. 1:**
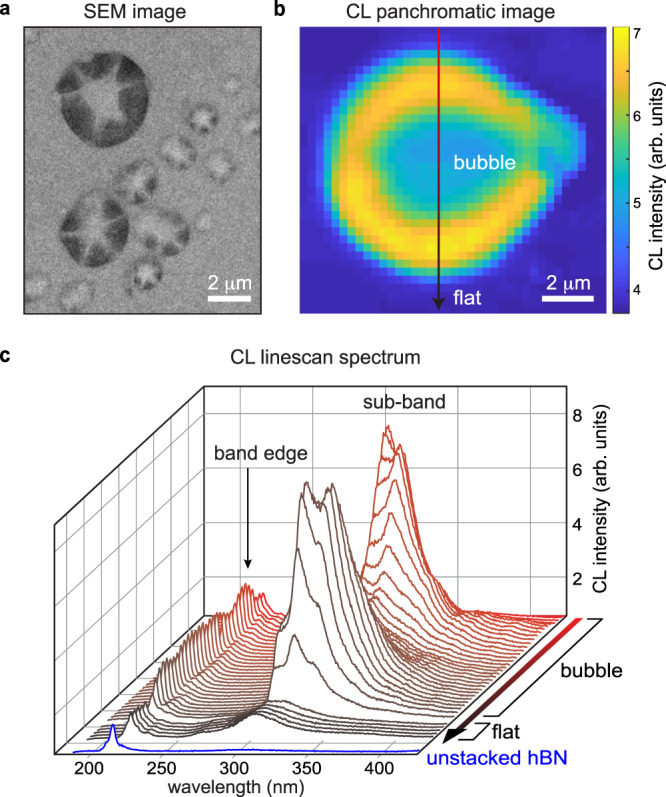
Cathodoluminescence from multilayer hBN bubbles. **a** SE2 image of representative hBN (100 nm)/hBN (50 nm) bubbles measured at 5 kV. **b** Panchromatic SEM-CL intensity map (5 kV) of a bubble in a suspended hBN (100 nm)/hBN (150 nm) double-multilayer structure, forming an optical cavity. The arrow shows the line scan direction for (**c**). **c** CL line scan across the bubble along *y* direction. For reference, the band-edge CL spectrum of an unstacked bottom hBN multilayer is shown in blue.

Stacks of multilayer hBN/multilayer hBN (referred to as hBN double-multilayers) were formed by mechanical exfoliation from high-quality single crystal hBN^20^, followed by vertical stacking onto an Si/SiO_2_ substrate using a modified dry transfer method^6^. During this process, surface adsorbents are trapped between the two stacked multilayers, forming bubbles at the interface within the double-multilayer. The samples were then annealed at 250 °C for 6 h, during which the initial bubbles merged to create fewer but larger bubbles^25^. The annealing time was selected to reach an equilibrium^26^ after which no further changes in size or shape of bubbles were observable. After the annealing, the bubbles formed between the two multilayers are mostly circular (Fig. 1a), unless they are near a line defect or an edge. In Fig. 1a, the sixfold pattern in the type II secondary electron (SE2) image is observed due to the presence of a radially symmetric strain field combined with electron channeling effects. As the electron beam scans across the bubble, the relative tilt of the lattice with respect to the beam changes due to the bubble curvature (Supplementary Fig. S1). Because the penetration depth and spreading of the electron beam depends on the orientation of the incident beam with respect to the lattice^27^, the yield of backscattered electrons—and hence intensity of the SE2 signal—varies across the bubble following the symmetry of the imaged crystal^28^.

### Enhanced luminescence from the bubbles

To study optical properties of the bubbles, we use cathodoluminescence (CL) attached to either a scanning electron microscope (SEM) or a scanning transmission electron microscope (STEM). CL utilizes a high energy electron beam to excite optical transitions in a material throughout the interaction volume, therefore enabling high resolution optical characterization of nanomaterials such as encapsulated monolayer TMDs and multilayer hBN^15,29,30^. Prior to the CL measurements, stacked hBN samples were transferred and suspended on a transmission electron microscopy (TEM) grid^31^ to eliminate any effect of the Si/SiO_2_ substrate.

A panchromatic SEM-CL intensity map of a representative hBN bubble excited using 5 kV electrons and integrated across all the photon energies (Fig. 1b) shows a strong and localized emission with a circular interference pattern indicative of radially-symmetrical light interference behavior. A CL linescan across the bubble (Fig. 1c) shows dramatic changes both in the CL intensity and the dominant optical transitions, compared to an unstacked bottom hBN multilayer and the flat hBN/hBN double-multilayer region without bubbles. From all three regions, we observe intrinsic near-band-edge excitonic recombination at 215 nm (5.77 eV), demonstrating the high crystalline quality of the hBN^20^ even after stacking and transferring. The spectrum from the flat double-multilayer exhibits an additional symmetric peak at 320 nm (3.9 eV) due to the atomic misalignment between the two multilayers^15^ with a measured twist angle of 29°. Finally, a strong emission spanning 300–400 nm (3–4 eV) is observed only in the bubble region. This sharp and asymmetric emission with zero-phonon line centered at 303 nm (4.09 eV) and phonon replicas with a constant spacing of 180 meV (Supplementary Fig. S2) indicates a distinct origin compared to the luminescence from the flat double-multilayer. The emission band at 300–400 nm in hBN has been previously attributed to carbon-related defects, such as substitutional carbon impurities^20,32^, which we later show is likely the origin of the emission in the bubble region. As the bubble effectively acts as a radially-symmetric optical cavity, the constructive and destructive interference of the emitted light that depends on the distance between two multilayers within the bubble result in the radially-dependent of the CL intensity across the bubble. For example, in Fig. 1b, CL from hBN multilayers interferes constructively near the edge of the bubble and interferes destructively near the center, leading to the brighter edge. Interference patterns for bubbles of different sizes are shown in Supplementary Fig. S3. To elucidate the origin of the strong phonon-coupled luminescence and potentially control the cavity modes by changing the bubble size and shape, it is essential to understand the mechanical properties of the multilayer bubbles as well as the nature of the material trapped inside.

### Mechanical properties of multilayer bubbles

**Fig. 2:**
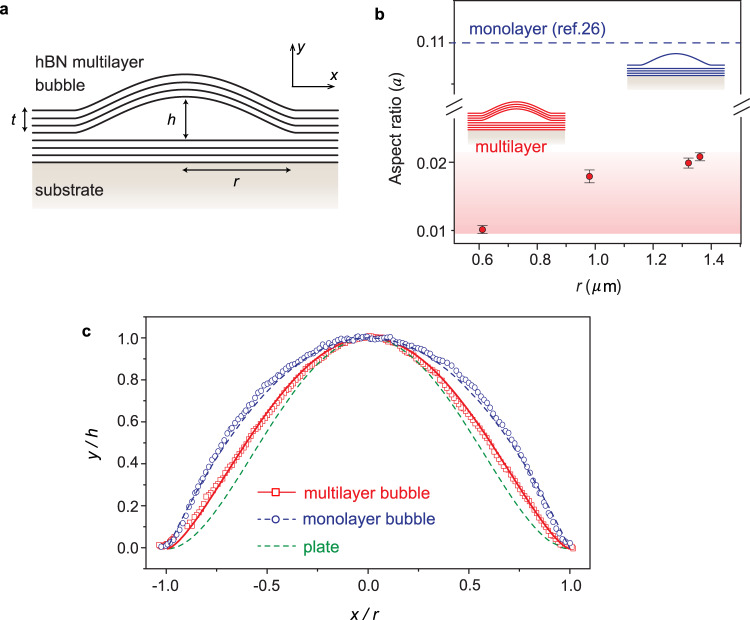
Multilayer vs. monolayer bubble geometry. **a** Schematic of a bubble formed between two multilayers of hBN/hBN on a Si/SiO_2_ substrate. The radius and height of the bubble are *r* and *h* respectively, and the thickness of the top multilayer is *t*. **b** The aspect ratio *a* as a function of the bubble radius *r* measured from an hBN/hBN multilayer bubble ($t=70\ {{{{{\rm{nm}}}}}}$, Supplementary Fig. S5). The dashed line indicates the aspect ratio reported previously for hBN monolayer bubbles, which is a constant independent of *r*^26^. The shaded region shows the experimentally measured aspect ratio for multilayer bubbles, which is *r*-dependent and significantly lower than for the monolayer counterpart. Error bars represent one standard deviation above and below the mean. **c** Experimental deflection profiles experimentally measured from a representative hBN/hBN multilayer bubble (squares) and a hBN/hBN monolayer bubble (circles). The red solid curve fitted to Eq. (1) yields $\alpha=1.5$. Analytic solutions are also shown for the elastic plate (dashed green line, $\alpha=2$) and membrane (dashed blue line, $\alpha=1$) models.

Multilayers of vdW layered materials are expected to show different bending behavior compared to a monolayer since the bending rigidity cannot be neglected in the case of multilayers, unlike the case of a single layer. We now show that the bubbles buried inside vdW multilayers can be used as an experimental tool to extract the bending rigidity of vdW multilayers. As shown in Fig. 2a, the bubble geometry is described by the bubble height *h* and radius *r*, and the aspect ratio $a=h/r$ determines the maximum strain at the center of a bubble, ${\varepsilon }_{0}\propto {a}^{2}$,^33^ which can also be measured by Raman spectroscopy (Supplementary Fig. S4). As the stack is supported by the Si/SiO_2_ substrate, only the top hBN multilayer is expected to deform. The bubble dimensions and shapes as well as the thickness of multilayers were measured using atomic force microscopy (AFM) in tapping mode. For a bubble with the thickness of the top multilayer *t* = 70 nm, we observe that the bubble aspect ratio varies between 0.01 and 0.02 (Fig. 2b), which is an order of magnitude smaller than the reported values for hBN monolayer bubbles (0.11 is measured for monolayer hBN on hBN^26^). Moreover, the aspect ratio is a function of the radius *r*, unlike the aspect ratio of monolayer bubbles that is independent of *r*^7,26,34,35^.

From Fig. 2b, it can be seen that in addition to the magnitude of the aspect ratio, the deflection profile (i.e., the shape) of the bubbles is different for the multilayer bubbles compared to monolayer bubbles (Fig. 2c). The deflection profile determines the strain distribution from the bubble center to the edge^33,36^ and can be expressed as follows^37^:

1 $$\frac{y(x)}{h}={\left(1-\frac{{x}^{2}}{{r}^{2}}\right)}^{\alpha }$$

where *x* is the distance from the center of the bubble and *y*(*x*) is the deflection. In a conventional plate with a finite thickness, the exponent $\alpha=2$, whereas $\alpha=1$ is assumed for membranes with negligible thickness^37,38^. The fundamental difference between the plate and membrane theory is in the assumed sample thickness. In the classical plate theory, a finite thickness of a plate results in a finite bending rigidity, whereas the membrane analysis assumes negligible thickness and consequently ignores the bending rigidity^36^. Due to the extreme thinness of 2D monolayers, the membrane model ($\alpha=1$) has been successfully applied for monolayer bubbles^33,34,36,39^, and is confirmed in our control case of an hBN monolayer (blue circles in Fig. 2c). However, in the case of a representative hBN multilayer bubble (red squares in Fig. 2c), fitting our experimental results yields $\alpha=1.5$, which is between these two theoretical limits. This result indicates that multilayer deformation cannot be fully explained by the membrane theory used for individual monolayers because of the non-negligible bending rigidity of multilayers, nor by the classical plate theory used for conventional thin films due to the weak vdW interaction between layers.

As the bending rigidity is a dominant parameter that governs the out-of-plane deformation, we next calculate how it determines the aspect ratio *a* in the case of multilayer compared to a single monolayer. We do this by considering a theoretical model of a multilayer bubble at equilibrium where its total energy (${E}_{{tot}}$) is minimized with respect to its height and radius^26,33,36^. ${E}_{{tot}}$ can be described by four energy terms when no external strain is applied: (i) in-plane elastic energy (${E}_{{el}}$), (ii) bending energy (${E}_{{bend}}$), (iii) adhesion energy between the bent multilayer and the substrate multilayer (${E}_{{adh}}$), and (iv) free energy of the material inside the bubble (${E}_{b}\left(V\right)$):

2 $${E}_{{tot}}={E}_{{el}}+{E}_{{bend}}+{E}_{{adh}}+{E}_{b}\left(V\right)$$

Each energy term can be further described as ${E}_{{el}}={{{{{{\rm{c}}}}}}}_{1}Y{h}^{4}/{r}^{2}$, ${E}_{{bend}}={{{{{{\rm{c}}}}}}}_{2}\kappa {h}^{2}/{r}^{2}$, ${E}_{{adh}}={{{{{{\rm{c}}}}}}}_{3}\gamma {r}^{2}$, $\partial {E}_{b}\left(V\right)/\partial V={-P}$, $V={{{{{{\rm{c}}}}}}}_{4}h{r}^{2},$ where *Y* is in-plane stiffness, *κ* is bending rigidity, *γ* is adhesion energy, *V* is the volume of the bubble, *P* is the pressure inside the bubble, and ${{{{{{\rm{c}}}}}}}_{1},{{{{{{\rm{c}}}}}}}_{2},{{{{{{\rm{c}}}}}}}_{3},{{{{{{\rm{c}}}}}}}_{4}$ are constants^26^. The aspect ratio *a* is then calculated by minimizing ${E}_{{tot}}$ with respect to *h* and *r*.

For a monolayer, the bending energy term is neglected ($\kappa=0$) due to its extreme thinness (the membrane model), and the aspect ratio is simplified to Eq. (3) below.^26^ Details of the calculation are provided in Supplementary Note 1.

3 $$a={\left(\frac{\gamma }{{c}_{1}{Y}_{{mono}}}\right)}^{1/4}$$

Equation (3) shows that the aspect ratio of a monolayer bubble depends only on the ratio between the adhesion and in-plane elastic energies of the 2D crystal, resulting in a universal aspect ratio for a specific material that is independent of the bubble size. For a monolayer of hBN, the aspect ratio can be calculated using the values of the in-plane stiffness for a monolayer ${Y}_{{mono}}\, \approx \, 22\left(\pm 6\right){{{{{\rm{eV}}}}}}{\dot{{{{{{\rm{A}}}}}}}}^{-2}$, $\gamma \, \approx \, 0.005\,{{{{{\rm{eV}}}}}}{\dot{{{{{{\rm{A}}}}}}}}^{-2}$ and ${c}_{1} \, \approx \, 1.55$,^26,40^ which leads to the aspect ratio $a=0.110$ ($\pm 0.008$), consistent with the experimental reports^26^. The values of ${Y}_{{mo}{no}}$ reported in other literature^18,41^ are within 20% of the value used here, which does not result in a substantial change in the aspect ratio *a*. The parameters used here are summarized in Supplementary Table 1.

We next extend the model to the case of a multilayer, where the bending rigidity cannot be neglected ($\kappa \, \ne \, 0$):

4 $$a={\left(\frac{\gamma {h}^{2}}{{{{{{{\rm{c}}}}}}}_{1}{Y}_{{multi}}{h}^{2}+{{{{{{\rm{c}}}}}}}_{2}\kappa }\right)}^{1/4}$$

**Fig. 3:**
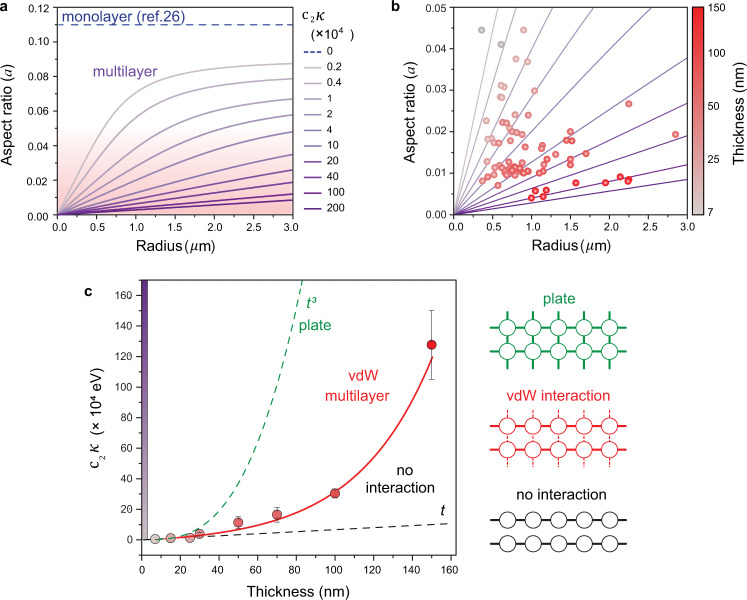
Mechanical properties of multilayer hBN bubbles. **a** Calculated aspect ratio *a* as a function of bubble radius for various values of the geometry-dependent bending rigidity. The shaded area indicates the region magnified in **b** where the aspect ratio is an approximately linear function of radius. **b** Experimentally measured aspect ratio of hBN/hBN multilayer bubbles (solid circles) as a function of the bubble radius and the multilayer thickness (represented by the symbol color). The solid lines are theoretically calculated values shown in **a**. **c** The experimentally determined bending rigidity. Error bars represent one standard deviation above and below the mean or are smaller than the symbols and the solid red curve is a guide to the eye. Two dashed curves show $\sim {t}^{3}$ and ~*t* dependency, which correspond to the case of an elastic plate and multilayers with no interlayer interaction, respectively. The schematics show atomic configurations for each case with different interlayer interaction. The color gradient along the y axis corresponds to bending rigidity in (**a**).

${Y}_{{multi}}$ is the in-plane stiffness of the multilayers (${Y}_{{multi}}={Y}_{{mono}}\bullet t$). We note that *κ* also scales with thickness, however, we do not assume a specific functional dependence at this point and allow ${\kappa}$ ∼ ${t}^{x}$. *γ* is independent of the thickness and ${{{{{{\rm{c}}}}}}}_{1}$ and ${{{{{{\rm{c}}}}}}}_{2}$ are dimensionless coefficients that depend on the in-plane and out-of-plane displacement, respectively. Therefore, only ${{{{{{\rm{c}}}}}}}_{2}$ varies with the deflection profile of the bubbles, and hence their thickness. As *κ* and ${{{{{{\rm{c}}}}}}}_{2}$ both change with thickness, the term ${{{{{{\rm{c}}}}}}}_{2}\kappa$, the geometry-dependent bending rigidity, provides a more comprehensive relationship between the aspect ratio and the thickness by capturing the combined effect of bending rigidity and deflection profile on the aspect ratio. Figure 3a shows the calculated aspect ratios as a function of the bubble radius *r* for different values of the geometry-dependent bending rigidity ${{{{{{\rm{c}}}}}}}_{2}\kappa$ from 0 to 200 $\times {10}^{4}{{{{{\rm{eV}}}}}}$. As shown in Fig. 3a, for non-zero ${{{{{{\rm{c}}}}}}}_{2}\kappa$, the aspect ratio of multilayer bubbles increases with their radius. Furthermore, as the ${{{{{{\rm{c}}}}}}}_{2}\kappa$ increases, the slope of the curve decreases, extending the linear regime in which the aspect ratio is approximately a linear function of the radius and the effect of the thickness dependence of ${Y}_{{multi}}$ on aspect ratio is negligible (Supplementary Fig. S6). An important consequence of this analysis is that unlike monolayer bubbles, where the strain is consistent, the aspect ratio and strain of multilayer bubbles can be engineered systematically through the radius and the multilayer thickness. For example, a change in strain of an order of magnitude can be induced by changing the bubble radius from 0.2 μm to 0.3 μm for *t* = 7 nm, or by changing the multilayer thickness from 10 nm to 30 nm for $r=0.3\,{{{{{\rm{\mu }}}}}}{{{{{\rm{m}}}}}}$. Our experimental results are in excellent agreement with the theoretical prediction: the aspect ratio of hBN bubbles decreases with increasing multilayer thickness and for each thickness, the aspect ratio increases with radius (Fig. 3b, Supplementary Fig. S7). It is worth noting that this model is applicable to other vdW layered materials, as we demonstrate for WS_2_ in Supplementary Fig. S8.

By comparing the experimental data with the calculated results for different ${{{{{{\rm{c}}}}}}}_{2}\kappa$, we can now extract the ${{{{{{\rm{c}}}}}}}_{2}\kappa$ that corresponds to each thickness, which enables us to unravel the relationship between thickness and bending rigidity, as shown in Fig. 3c. In the classic linear plate theory, bending rigidity is proportional to thickness cubed ($\kappa \sim {t}^{3}$)^37^, whereas in the case of free shear interaction (i.e., no interaction) between adjacent layers, bending rigidity is linearly proportional to thickness ($\kappa \sim t$)^18^. These are the two limiting cases where the first ignores the possibility of sliding (infinite friction of sliding), whereas the second ignores interlayer interaction (zero friction of sliding). In the case of vdW layered materials, however, the thickness dependence of the bending rigidity is between the two cases, as we show in Fig. 3c and also corroborated by other studies^18^. This behavior can be understood by considering the anisotropic in-plane and out-of-plane mechanical properties of an individual layers^18,42–44^ and weak vdW interactions between the layers, which may subject the vdW multilayer to interlayer sliding.

### Electron beam-induced modulation of the bubble geometry

**Fig. 4:**
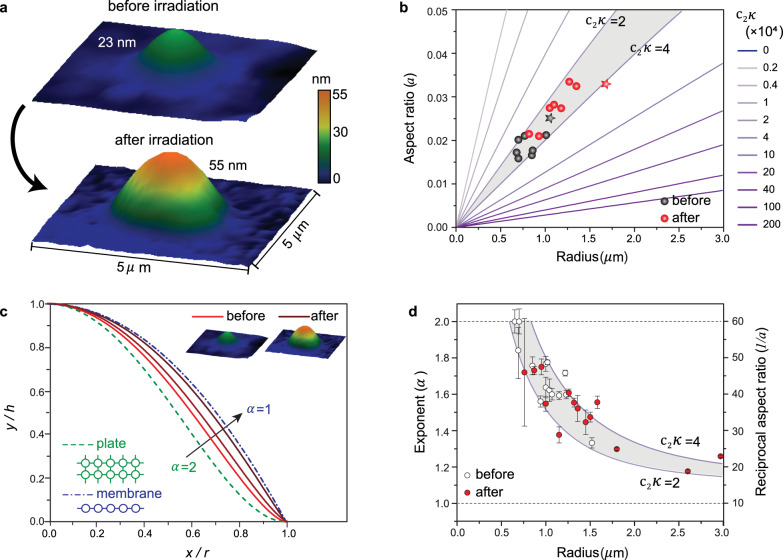
Electron-beam-induced modulation of hBN bubbles. **a** 3D AFM map of a multilayer bubble ($t=50 \, {{{{{\mathrm{nm}}}}}}$) before and after electron beam irradiation. vdW pressure of the bubbles are calculated in Supplementary Note 1. **b** Aspect ratio of the bubbles before (black) and after (red) irradiation with the theoretically calculated aspect ratio for different ${c}_{2}\kappa$ values in Fig. 2a. All data points lie between ${c}_{2}\kappa=2$ and ${c}_{2}\kappa=4$. The star symbol indicates the bubble shown in (**a**). **c** Fitted curves of the normalized deflection profiles of a representative multilayer bubble to Eq. (1). The deflection profiles of the same bubble are measured three times: before irradiation, $\alpha=1.53$ (red), after the first dose ($\alpha=1.36$, dark red) and after second dose ($\alpha=1.10$, darker red). Two dashed curves are the two limits shown in Fig. 1d. **d** The values of exponent (*α*) obtained by fitting the deflection profiles measured from pre-irradiated (black) and irradiated (red) bubbles in **b**. The reciprocal of theoretically calculated aspect ratio (1/*a*) is plotted, showing that the data points are still between ${c}_{2}\kappa=2$ and ${c}_{2}\kappa=4$. Error bars represent one standard deviation above and below the mean.

**Fig. 5:**
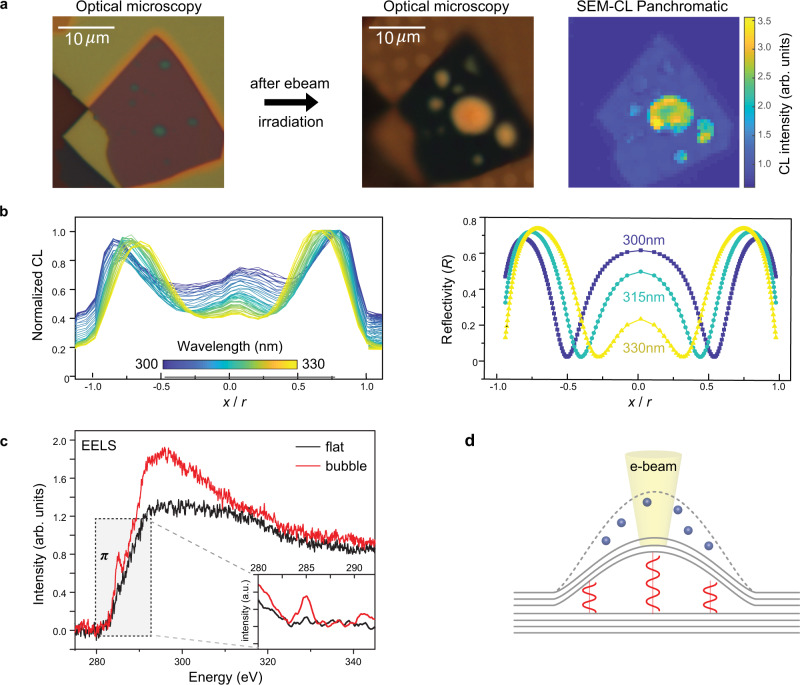
Origin of the enhanced cathodoluminescence from hBN bubbles. **a** Optical microscope image before (hBN (70 nm)/hBN (70 nm) on Si/SiO_2_) and after (suspended hBN/hBN) CL measurement, scale bar 10 *μm*, and SEM-CL panchromatic map at 5 kV. The STEM-CL map is shown in Supplementary Fig. S10. **b** Left: Normalized CL intensity at a specific wavelength (300–330 nm) across the bubble in Fig. 1. Right: Reflectivity at specific wavelengths (300, 315, 330 nm) calculated using a transfer matrix method across a bubble with $h=100$ nm assumed filled with polycarbonate. **c** EELS of carbon K edge peaks measured on the bubble and at a flat region nearby. The inset shows the spectrum in the shaded region after linear subtraction. **d** Schematic showing the expanding bubble and standing waves.

We next turn to the question of whether the bubble geometry can be modified, both to increase our understanding of the mechanical properties and to eventually tailor the multilayer optical properties. As mentioned previously, the bubbles are filled with polymeric materials such as hydrocarbons, air, or water. We find that this confinement of materials can be used to expand the bubble size using electron beam irradiation. The bubbles were irradiated inside an SEM, using electron energy between 1 and 10 kV, which is far below the threshold energy of hBN. Their geometry was subsequently measured by AFM (see Supplementary Note 2). Figure 4a shows 3D AFM maps of a representative hBN bubble before and after irradiation at 3 kV, demonstrating a 5× volume increase and 1.6× increase in the aspect ratio. We suggest that decomposition of organic compound inside the bubble by the electron beam is a likely cause of the expansion and provide supporting evidence in Fig. 5 below. It is interesting to note that the aspect ratio still follows the theoretical model even after irradiation, as shown in Fig. 4b, indicating that Eq. (2) is still applicable and can be used to predict the geometry of the expanded bubbles. Measurements on multiple bubbles show that all the extracted values *κ* before and after irradiation remain similar ($2\le {{{{{{\rm{c}}}}}}}_{2}\kappa \le 4$), implying that the electron beam does not dramatically change the intrinsic mechanical properties (*Y*, *κ*, and *γ*) of the multilayer.

While the mechanical parameters are preserved, the deflection profiles show a transition between the two limits ($\alpha=1 \; {{{{{\rm{and}}}}}}\; \alpha=2$), approaching the membrane limit as the bubble expands (Fig. 4c), likely due to the increasing maximum strain^45^. We note a sharp change in curvature near the edge in the transition from $\alpha=2$ to $\alpha=1$, which can cause strain concentration at the edge (Supplementary Fig. S9). The deflection profiles from all bubbles in Fig. 4b were collected and the values of *α* obtained by fitting to Eq. (1) are shown in Fig. 4d. The exponent approaches 1 with increasing radius, and the reciprocal of the aspect ratio 1/*a* calculated by the theoretical model matches our experimental results. Therefore, in addition to its aspect ratio, the bubble shape can also be predicted by the model, which enables us to calculate both the strain at the bubble center and the strain distribution across the bubble including the stress concentration at the edge. This result broadens our understanding of vdW multilayer bending, since previous literature has suggested that the deflection profiles of multilayer bubbles collapse onto one curve ($\alpha=1$ or $\alpha=2$)^18^.

### Origin of the enhanced luminescence from the bubbles

By combining the tunability of the bubble geometry using an electron beam while simultaneously measuring optical properties using CL, we can examine the origin of the strong and localized optical emission shown in Fig. 1 and also obtain information regarding the composition of the material within the bubble. CL measurement at different accelerating voltages enables us to investigate the depth profile of optical emission of a sample because of the difference in electron penetration depth (Supplementary Fig. S10). We therefore irradiated the sample at 80 keV, measuring STEM-CL, then irradiated at 5 keV while measuring SEM-CL (Fig. 5a and Supplementary Fig. S10). Comparing the STEM-CL and SEM-CL panchromatic maps in Supplementary Fig. S10a and d, only SEM-CL shows strong emission from the bubble region, whereas the bubbles appear dark in STEM-CL. For the 5 keV electrons used in SEM, energy is absorbed mainly within the top hBN multilayer, whereas for the 80 keV electrons used in STEM, energy is absorbed throughout the entire sample thickness within a narrow (<5 nm) interaction volume (Supplementary Fig. S10b–f). The strong CL emission observed in the bubble region that is only measured at low accelerating voltage therefore originates from the top strained hBN multilayer. This is further confirmed by comparing CL measurement at 5 keV and 10 keV (Supplementary Fig. S11).

To further understand the origin and localization of this luminescence, we next focus on the optical interference and show that the bubbles are likely filled by organic compounds such as hydrocarbons. For the bubble in Fig. 1b, c, we show the measured CL peak intensity across the bubble (Fig. 5b, left) at several wavelengths in the range 300–330 nm. Since peak shifts of the zero-phonon line and its phonon replicas are not observed, we can attribute changes in emission intensity across the bubble to interference. We take advantage of our mechanical model to determine the shape and height of the bubble from the experimentally measured bubble radius ($r=4\,{{{{{\rm{\mu }}}}}}{{{{{\rm{m}}}}}}$) and layer thickness ($t=150\,{{{{{\rm{nm}}}}}}$). Based on this calculated shape ($h=100\,{{{{{\rm{nm}}}}}}$ and $\alpha=1.7$), we conduct transfer-matrix method reflection simulations^46^ that assume optical properties of several likely candidates: polycarbonate (Fig. 5c), air, and water (Supplementary Fig. S12). For these calculations, we measured the optical constants (refractive index and extinction coefficient) of the hBN multilayer using ellipsometry while the wavelength-dependent optical constants of polycarbonate^47^ and water^48^ were taken from literature. By comparing the experimental results with the simulations, the closest match is for polycarbonate, from which we conclude that the refractive index of the material inside the bubble is $n\ge 1.65$ at 300 nm. We note that this simulation is consistent with data from bubbles of different sizes (Supplementary Fig. S3). Taken together, these results suggest that organic compounds of density similar to polycarbonate fill the bubble. This material presumably originates from the dry transfer process. As polymeric materials including polycarbonate readily decompose under an electron beam by beam-induced ionization and dissociation, producing uncombined free carbon species^49^, this likely accounts for the electron beam-induced expansion.

We finally investigate the structural features of carbon inside the bubbles qualitatively by comparing electron energy loss spectroscopy (EELS) measured from the bubbles and from flat regions near the bubbles (Fig. 5c). The carbon K edge peak from the bubbles shows a clear feature of sp^2^ ^2^ bonding (285 eV) and a relatively narrow peak (295 eV), whereas the spectrum measured from flat regions shows only broad features that have been observed in amorphous carbon^50^. We speculate that this change in carbon bonding state may be caused by structural modification of the strained hBN multilayer due to incorporation of carbon into hBN.

## Discussion

An asymmetric luminescence with phonon replicas in the spectral range of 300–400 nm, similar to what we observe in the bubble region (Fig. 1c), has been reported previously in hBN^20,32^. It has been attributed to carbon-related defects such as the substitutional carbon impurities, which form deep impurity levels within the hBN band gap^20,32^. Based on our findings that (1) the emission originates from a deformed top hBN multilayer, (2) polycarbonate is confined inside the bubble and dissociated by electron beam irradiation resulting in modulation of the bubble geometry, and (3) the carbon bonding state changes inside the expanded bubble (Fig. 5d), we speculate that the trapped matter inside the bubble induces a structural modification of the strained hBN multilayer, resulting in strong and local luminescence from the bubble. Possible carbon doping is also consistent with the previous reports that show reduced radiation hardness of hBN under strain^51^ and electron beam-induced carbon doping in hBN^52^. Furthermore, the optical standing waves formed within the expanded bubble depend on its geometry (Supplementary Fig. S3), which can be calculated by our mechanical model and modulated by the electron beam.

Our results suggest that nanoscale bubbles can be created in a controlled manner with properties of interest for optoelectronic and photonic applications, for example photonic crystals formed by creating arrays of nanoscale bubbles where each component plays role as an ultraviolet emitter. As strain across the bubble enhances the incorporation of dopants, bubbles can also serve as an effective tool to study strain-related optical properties within a band gap. It is also possible to tune the emission wavelength further by changing the filling materials inside the hBN bubble: for example, Ni or Cu in hBN can result in luminescence in the visible regime^53,54^.

We have observed strong and localized luminescence in the ultraviolet region from the bubbles formed between two hBN multilayers and have suggested a model for this luminescence based on analysis of the mechanical characteristics of strained vdW multilayers. Because deformation of a vdW multilayer is dominated by its bending rigidity, in contrast to a monolayer, we show that bubbles formed in vdW multilayers show a distinct geometry both in aspect ratio and shape. The geometry of bubbles and the thickness-dependent bending rigidity can be calculated using the theoretical model developed here, which is a precondition for strain engineering. As a pathway to modify the geometry of the bubbles, we find that irradiation by an electron beam can decompose the trapped materials inside the bubbles, increasing their luminescence. These results open a route toward creation of strong ultraviolet optical emitters and suggest opportunities for designing cavities for luminescence utilizing the bending of vdW multilayers and confinement of materials within. Intentional introduction and confinement of other materials within bubbles made from various vdW multilayers may further expand the possibilities of this strategy to induce local luminescence at different frequency ranges.

## Methods

### Materials and stacking

Our starting materials were bulk crystals of hBN synthesized by the high-temperature-high-pressure method^20^ and bulk crystals of WS_2_ from HQ Graphene. hBN and WS_2_ multilayers were mechanically exfoliated onto an Si/SiO_2_ substrate by the scotch-tape method, then vertically stacked via a modified dry transfer method using a polymer stamp (polydimethylsiloxane (PDMS)/polypropylene carbonate (PPC)). For the PDMS mask, a solution of 20:1 ratio of Sylgard 184 prepolymer to curing agent was kept at ambient condition for ∼24 h. After oxygen plasma treatment of PDMS (18 W for 5 min), 15% of PPC in anisole was spin-coated at 3000 rpm on the PDMS. The solvent was removed by heating the PDMS/PCC mask at 160 °C for 10 min. The polymer stamp was contacted to a top multilayer for 1 min at 50 °C for pick-up and then dropped onto a bottom multilayer at 100 °C followed by increasing the temperature to 120 °C for 2 min. To remove PPC residue, the sample was immersed in acetone for 5 min and isopropanol for 10 s. The resulting double-multilayers were annealed at 250 °C in an Ar environment for 6 h.

### Topographic characterization

Height profiles of bubbles on Si/SiO_2_ substrate were obtained before and after electron beam irradiation using a Veeco Dimensions 3100 AFM operated in tapping mode with 300 kHz tip. A Zeiss Merlin high resolution SEM was used for electron beam irradiation with current density of 100–500 pA and dwell time of 25 ms/pixel (${10}^{4}{-}{10}^{5}{{{{{\rm{electrons}}}}}}/{{{{{{\rm{nm}}}}}}}^{2}$) at 1–10 kV.

### Optical characterization

For CL measurements the samples were transferred to a C-flat TEM grid (Protochips Inc., 200 mesh, 2 μm holes) by a wet transfer method using poly(methylmethacrylate) (PMMA, 495 K, A4 Microchem) and 1 M potassium hydroxide. STEM-CL was measured first at 80 kV (using a Gatan MonoCL 3+ attached to a JEOL 2011 TEM) followed by SEM-CL at 5 kV (Attolight^TM^ Allalin) with dwell time of 20 ms/pixel.

The refractive index n of an exfoliated hBN flake with a thickness of 5 nm was measured with a spectroscopic imaging nulling ellipsometer EP4 (Accurion Gmbh, Göttingen, Germany) in ambient conditions and at room temperature.

### Interference simulation

The regions of constructive and destructive interference in the hBN/hBN stacks were calculated using a conventional transfer matrix method calculation^46^, implemented in MATLAB. The reflection depends on the spacing *d* between two multilayers, and the interference pattern can be predicted by calculating the reflection coefficient (*R*) through each layer using established *n* and *k* optical constants from literature and ellipsometry (Supplementary Fig. S13).

### EELS measurement

STEM-EELS measurements with energy resolution ~0.3 eV were obtained at 200 kV using a probe-corrected Thermo Fisher Scientific Titan G3 60–300 kV with monochromator and a Gatan Continuum spectrometer. High energy-loss spectra were aligned using the zero-loss shift determined using the simultaneously collected low-loss spectra. The carbon K-edge background was fitted to a power law and subtracted from the raw spectrum. The effects of plural scattering were removed from the spectrum using the Fourier ratio method.

## Supplementary information

Supplementary Information

## Data Availability

The datasets generated and analyzed during this study are included in this paper and its Supplementary Information, and are also available from the corresponding author upon reasonable request.
