# Supplementary material for: Strong and Localized Luminescence from Interface Bubbles Between Stacked hBN Multilayers
Source: Nat Commun. 2022 Aug 25;13:5000. doi: 10.1038/s41467-022-32708-z (PMC9411575; doi:10.1038/s41467-022-32708-z)
Supplement: Supplementary file 1 — Supplementary Information [file 41467_2022_32708_MOESM1_ESM.pdf]

Supplementary Information

Strong and Localized Luminescence from Interface Bubbles Between Stacked hBN Multilayers

Hae Yeon Lee[1], Soumya Sarkar[2], Kate Reidy[1], Abinash Kumar[1], Julian Klein[1], Kenji Watanabe[3], Takashi Taniguchi[4], James M. LeBeau[1], Frances M. Ross[1], Silvija Gradečak[1,2]

[1]Department of Materials Science and Engineering, Massachusetts Institute of Technology, 77 Massachusetts Ave, Cambridge, MA 02141, USA

[2]Department of Materials Science and Engineering, National University of Singapore, 9 Engineering Drive 1, 117575 Singapore

[33]Research Center for Functional Materials, National Institute for Materials Science, 1-1 Namiki, Tsukuba 305-0044, Japan

[4]International Center for Materials Nanoarchitectonics, National Institute for Materials Science, 1-1 Namiki, Tsukuba 305-0044, Japan

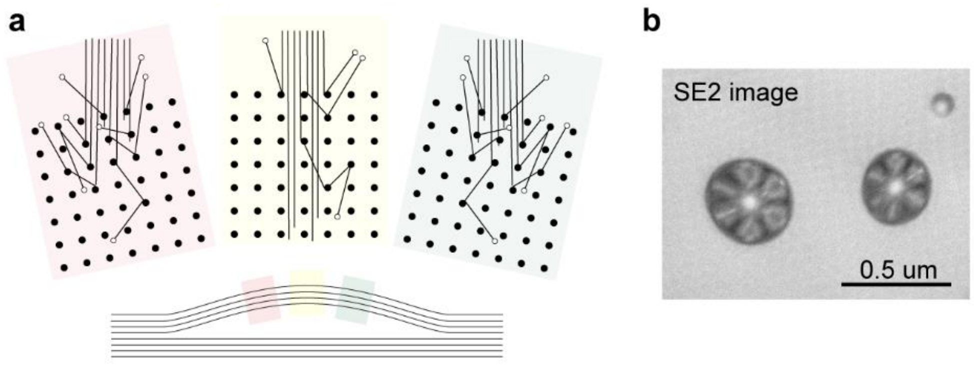

Supplementary Figure S1. Scanning electron microscopy (SEM) imaging of bubbles in van der Waals (vdW) multilayers. (a) The variation in electron backscattering as the crystal lattice changes its angle relative to the incident electron beam. (b) SEM image imaged by SE2 detector of bubbles between WS2 multilayers. The contrast is more prominent compared to the case of hBN due to its higher atomic number.

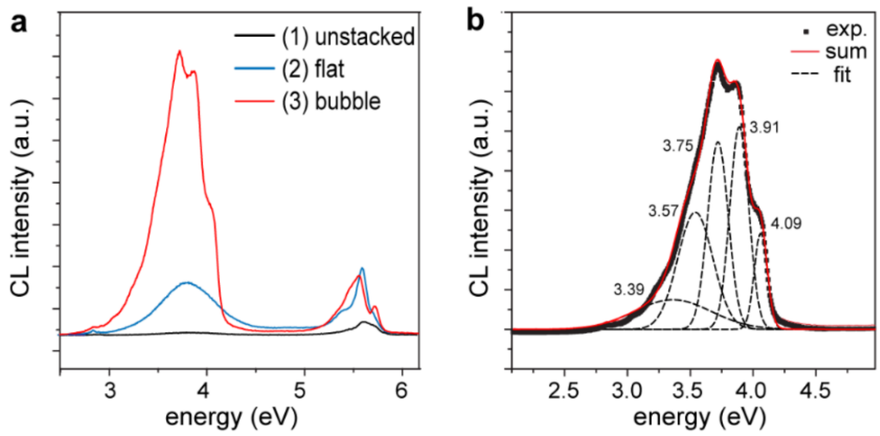

Supplementary Figure S2. Cathodoluminescence (CL) spectra and deconvolution of sub-bandgap emission band. (a) CL spectra measured at 5 kV from (1) unstacked hexagonal boron nitride (hBN) multilayer, (2) flat hBN/hBN double-multilayer, and (3) at the location of a bubble formed between two hBN multilayers as shown in Figure 1c in the main text. (b) Phonon-

coupled luminescence in the near-ultraviolet region (3 – 4 eV) measured from multilayer bubbles (black dots) is deconvoluted to multiple peaks (dashed line) centered at 4.09 (zero-phonon line), 3.91, 3.75, 3.57, 3.39 eV (phonon replicas) with a constant spacing (180 meV). The sum of deconvoluted peaks is shown in red. Those multiples peaks are assigned to single radiative recombination process as they all exhibit a single decay curve with characteristic decay time of 1.1 ns (*1*).

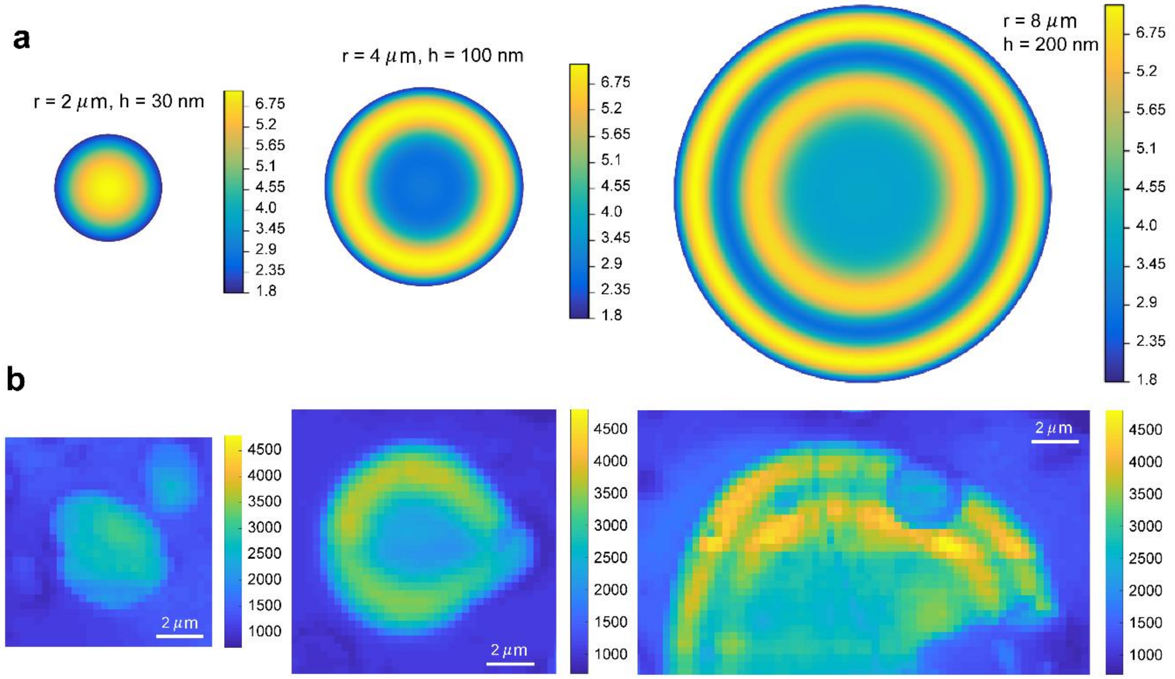

Supplementary Figure S3. Interference pattern of bubbles of different size. (a) Simulated results of bubbles of radius 2,4, and 8 μm, which is 30, 100, 200 nm high, respectively. Bubbles are formed between 150 nm thick top hBN multilayer and 100 nm thick bottom hBN multilayer. (b) Experimentally measured panchromatic CL map of bubbles of radius 2,4, and 8 μm.

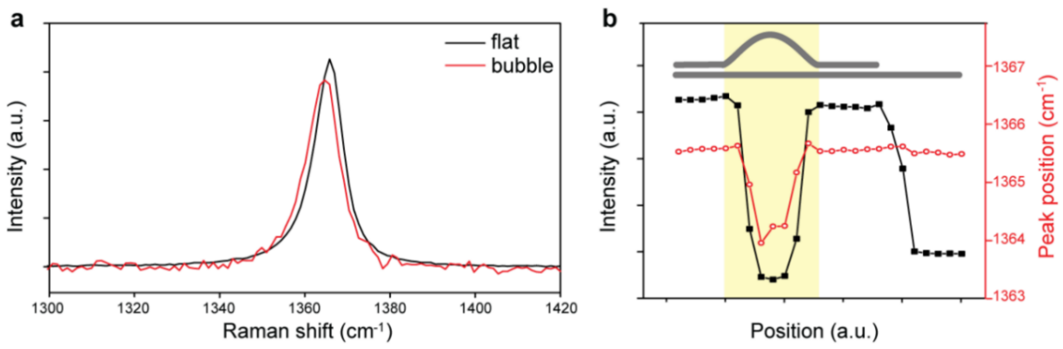

Supplementary Figure S4. Raman spectra measured across the bubble. (a) Raman spectra measured from flat double-multilayer and bubble regions. The Raman peak redshifts due to the strain in the bubble. (b) Raman intensity and peak position measured across the bubble (yellow region), where schematic shows the bubble position. The peak shift due to strain is maximized at the center of the bubble.

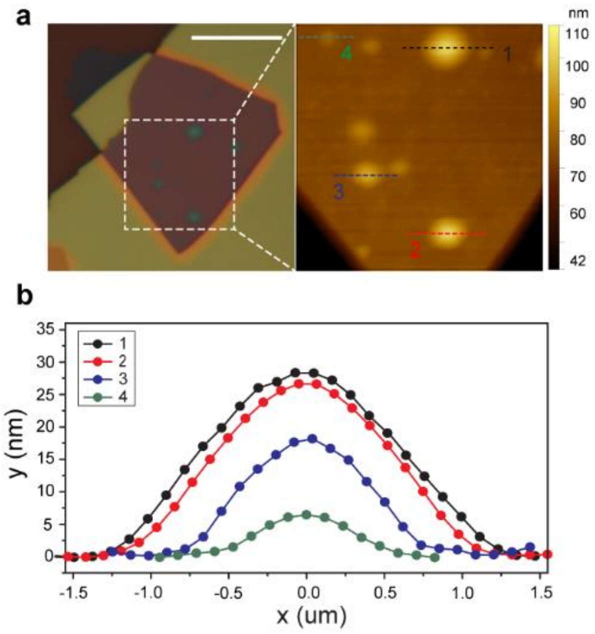

Supplementary Figure S5. Height profile measurement of bubbles in hBN/hBN. Bubbles in hBN/hBN ($t = 70\ nm$, Figure 2b in the main text) on Si/SiO_2. (a) Optical microscope image of

stacked hBN double-multilayer (scale bar 10 μm) and height map of dashed area measured by atomic force microscopy (AFM). (b) Height profiles along the bubbles indicated in the AFM map. The aspect ratio of each bubble is calculated as shown in Figure 2b in the main text.

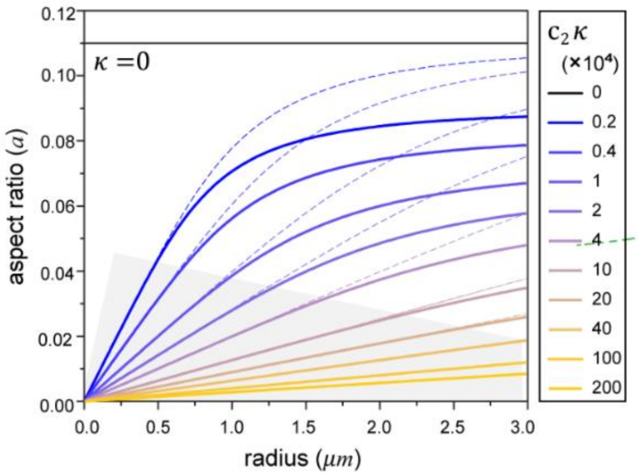

Supplementary Figure S6. Effect of thickness dependence of Y_{multi}on aspect ratio.

Calculated aspect ratio as function of bubble radius with various values of geometry-dependent bending rigidity $c_2\kappa$ when Y_{multi} is independent of the thickness (dashed lines) and when Y_{multi} linearly depends on the thickness (solid lines). The shaded area indicates the linear regime where experimental data are positioned (see Figure 2b). Both Y_{multi} and $c_2\kappa$ depend on the thickness of the multilayer even though their dependencies are different. Y_{multi} is linearly proportional to the thickness ($\sim t$) whereas the dependency of $c_2\kappa$ on the thickness is in between $\sim t$ and $\sim t^3$ as elaborated in the main text. Therefore, when $c_2\kappa$ varies from 0 to 200×10^4 eV, the value of Y_{multi} will change at the same time. We find that in the linear regime, the dependency of Y_{multi} on the thickness does not affect the aspect ratio significantly.

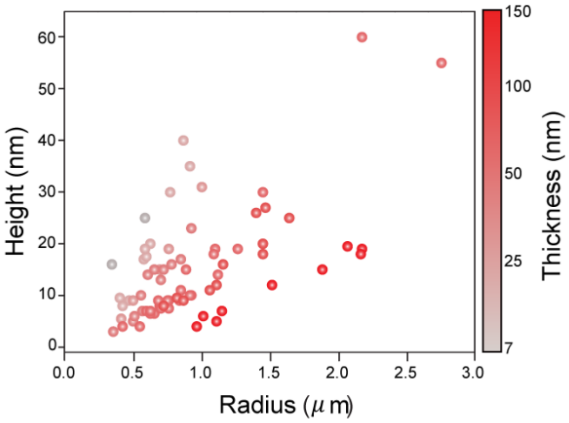

Supplementary Figure S7. Height of bubbles depending on the radius of bubbles and thickness of multilayer. Experimentally measured height of hBN/hBN multilayer bubbles (solid circles) shown in Figure 3b in the main text as a function of the bubble radius and the multilayer thickness (represented by the symbol color).

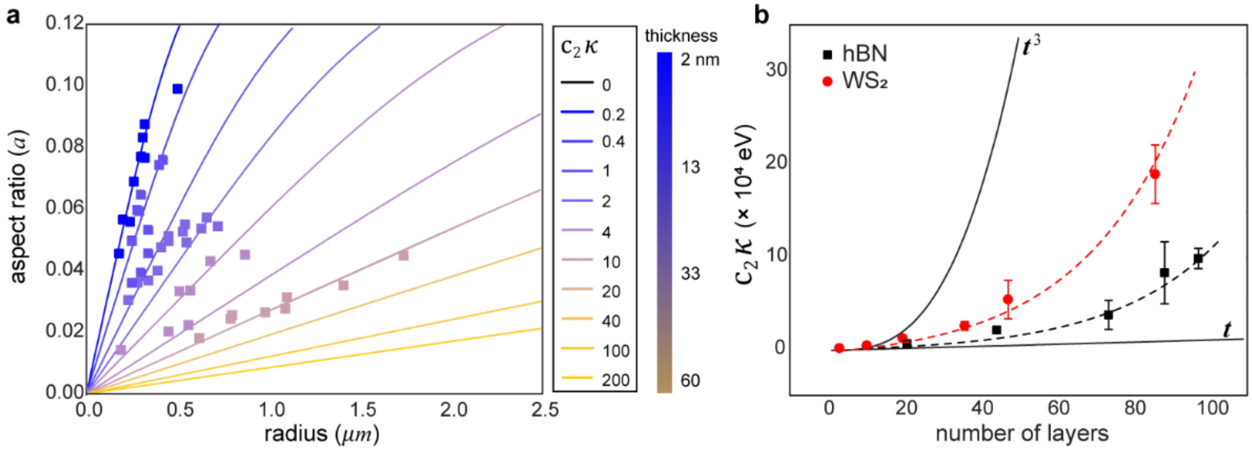

Supplementary Figure S8. Aspect ratio and bending rigidity analysis for WS_2. (a) Calculated aspect ratio of WS_2 multilayer bubbles as a function of radius with various values of $c_2\kappa$ (solid lines) and experimentally measured aspect ratio (squares). Multilayer bubbles formed by WS_2 multilayers of thickness 2 – 60 nm were measured and the thickness is represented by the

symbol color. The experimental data lie in the linear regime. $Y \approx 11.2\ eV/\dot{A}^2$, $\gamma \approx 0.015\ eV/\dot{A}^2$ and $c_1 \approx 1.55$ (*2*) are used for the calculation, which provides a value of $h/r = 0.17$ for monolayer bubbles. (b) $c_2\kappa$ of WS_2 multilayer extracted from (a) in comparison to that of hBN in main text, showing a larger bending stiffness of WS_2 compared to that of hBN, consistent with literature (*3*). The stiffer WS_2 monolayer can be understood by considering that it is composed of three atomic layers whereas the hBN monolayer has a single atomic thickness. The error bars represent one standard deviation above and below the mean.

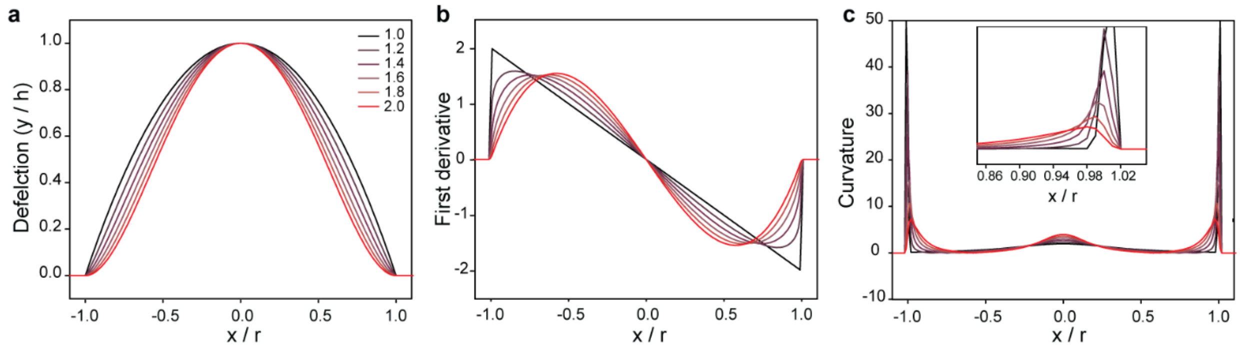

Supplementary Figure S9. Sharp changes in curvature of bubble near the edge. (a) Deflection profile with various values of α in the range 1.0 – 2.0 in the equation $\frac{y(x)}{h} = (1 - \frac{x^2}{r^2})^{\alpha}$. (b) First derivative of the deflection profile. (c) Curvature of the deflection profile. The curvature near the edge ($x/r \sim \pm 1.0$) significantly changes with α.

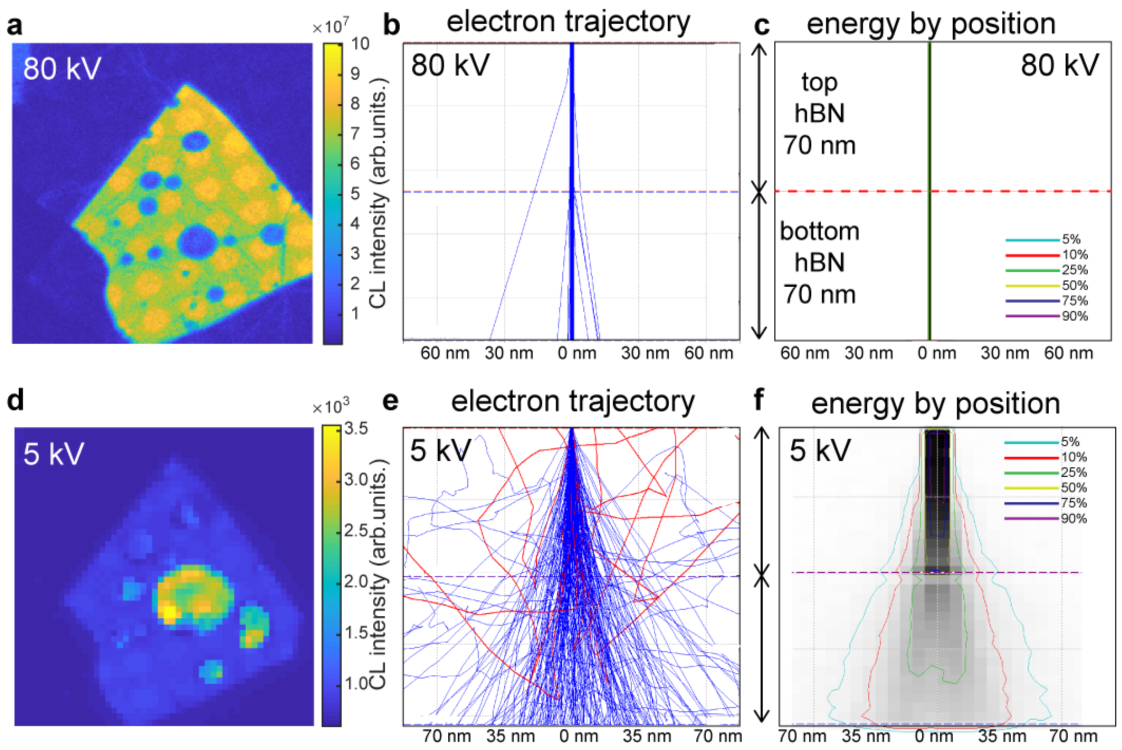

Supplementary Figure S10. Comparison between STEM-CL and SEM-CL. CL panchromatic map and electron beam interaction of an hBN double-multilayer suspended on a TEM grid at different accelerating voltage. (a) Panchromatic STEM-CL map of hBN (70 nm) /hBN (70 nm) double-multilayer measured at 80 kV. Luminescence from the bubbles is negligible compared to the flat region. (b) Cross-section view of Monte Carlo electron trajectories in the sample with an 80 kV incident beam, simulated using CASINO v.2.5.1 (*4*). (c) Spatial distribution of energy absorbed at 80 kV with energy contour lines. (*e.g.*, 10% line is the frontier between an area containing 90% of the absorbed energy and the rest of the sample.) The gray shading shows the density of absorbed energy. The 80 kV electrons penetrate the suspended double-multilayer and energy is absorbed homogeneously throughout the entire thickness within a narrow (< 5 nm) interaction volume. (d) Panchromatic SEM-CL map at 5 kV. The CL map shows strong luminescence from the bubbles, as in Figure 1 in the main text. (e-f) same as (b-c)

but at 5 kV. The interaction volume broadens and energy is absorbed mainly inside the top hBN multilayer. CL measurement at different accelerating voltages thus enables to investigate the depth profile of optical emission of the sample, as the spatial distribution of absorbed energy depends on the voltage. The bubble luminescence is only effectively observed for 5 kV SEM-CL measurements when most of the charge carriers are generated close to the surface, in contrast to STEM-CL. This implies that the optical emission from the bubble originates from the top hBN multilayer. Since low kV electrons have stronger interaction with the sample, we exclude beam-induced effects on this conclusion by first investigating the bubbles using STEM-CL (80 kV) and then SEM-CL (5 kV). The 80 kV measurement was then repeated to verify that no significant emission caused by irradiation of the bubble from the low kV measurement is observed from the bubbles. We note that no CL signal was detectable for hBN multilayer of less than 10 nm due to reduced interaction volume.

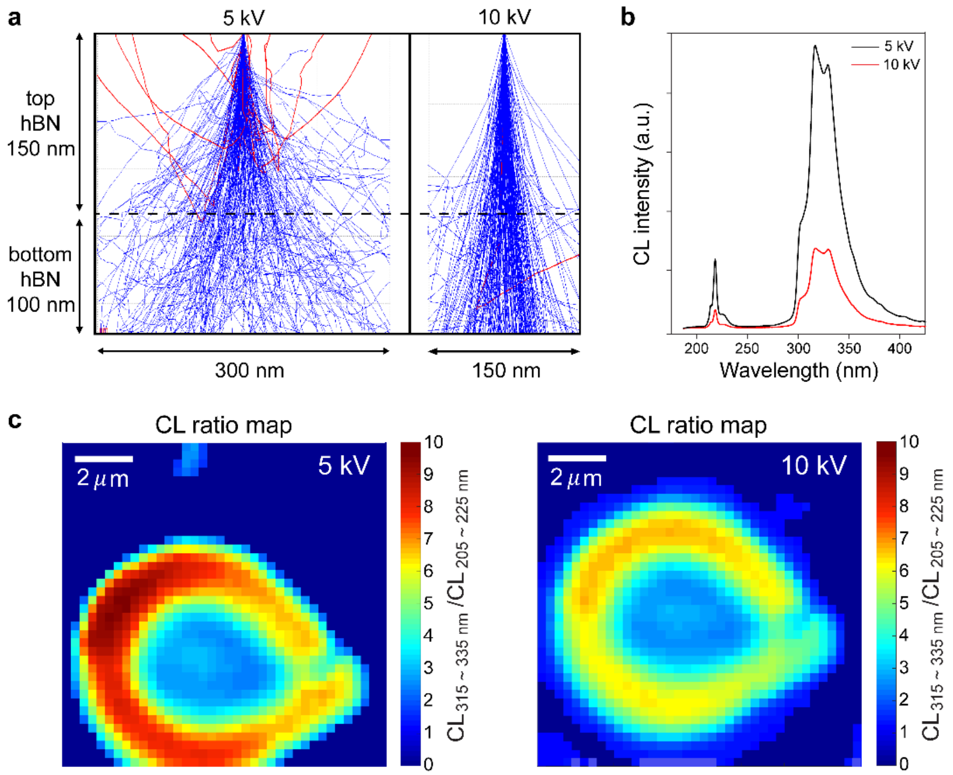

Supplementary Figure S11. Comparison between SEM-CL measurement at 5 kV and 10 kV. (a) Cross-section view of Monte Carlo electron trajectories in the sample with 5 and 10 kV incident beam, simulated using CASINO v.2.5.1. (b) CL spectra measured from the bubble at 5 and 10 kV. (c) CL intensity ratio map ($\frac{CL\ intensity_{315\sim335\ nm}}{CL\ intensity_{205\sim225\ nm}}$) at 5 and 10 kV. As discussed in Figure S10, the contribution of bottom multilayer to CL would increase with accelerating voltage due to the difference in penetration depth/interaction volume. Therefore, the contribution of each layer to CL can be analyzed by comparing band edge emission (215 nm) and sub-bandgap emission (300 – 400 nm). The CL intensity ratio ($\frac{CL\ intensity_{315\sim335\ nm}}{CL\ intensity_{205\sim225\ nm}}$) decreased at 10 kV, which indicates that the bottom multilayer is not responsible for the sub-bandgap emission but only for the band edge emission, which supports that the sub-bandgap emission is mainly from the top hBN multilayer.

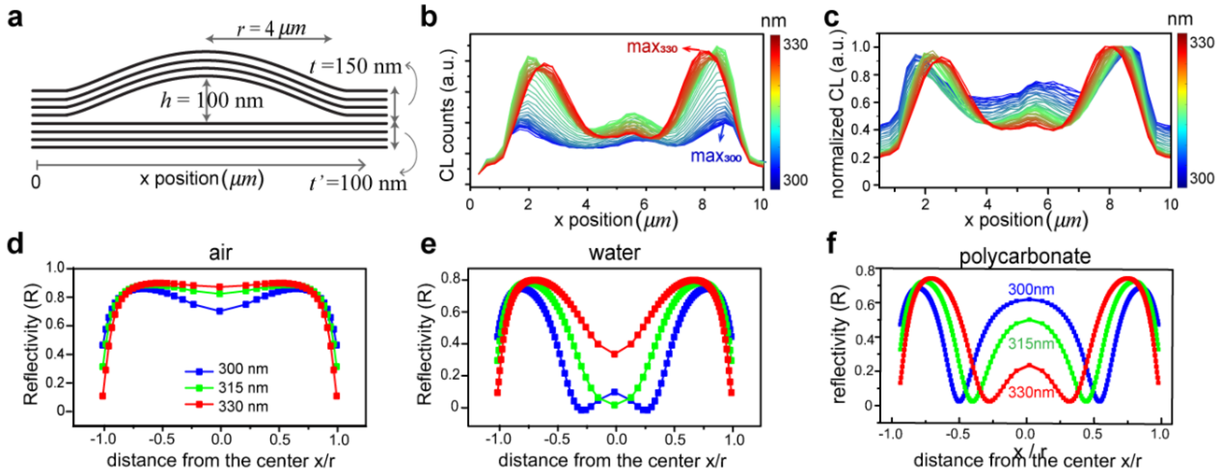

Supplementary Figure S12. Optical simulation of bubbles filled with different materials. Interference simulation of the multilayer bubble ($r = 4\ \mu m, h = 100\ nm, t = 150\ nm, t' = 100\ nm$) filled with different materials: polycarbonate, air, or water. (a) Schematic of the multilayer bubble with relevant parameters. As shown in Supplementary Fig.S10 and S11, CL is

emitted from the top multilayer and some of it is reflected by the bottom multilayer. The reflection depends on the spacing *d* between two multilayers and the interference pattern can be predicted from the reflection coefficient (R) through each layer (the top multilayer, the material inside the bubble, and the bottom multilayer) and the optical constants (refractive index and extinction coefficient). The regions of constructive and destructive interference in the hBN multilayer bubble structures can then be calculated using a conventional transfer matrix method using MATLAB. (b) Experimentally measured CL intensity at several wavelengths (I_λ) across the bubble in the *x*-direction. (c) CL intensity normalized by the maximum intensity at each wavelength (I_λ/max_λ). (d-f) Reflectivity calculated by interference simulation when the bubble is filled with (d) air (e) water, and (f) polycarbonate. Optical constants of the hBN multilayer are measured using ellipsometry and the polycarbonate (*5*) and water (*6*) values are from previous literature. Polycarbonate can remain at the interface after stacking multilayers especially when the top hBN multilayer does not fully cover the bottom hBN multilayer as polycarbonate directly contacts the bottom multilayer.

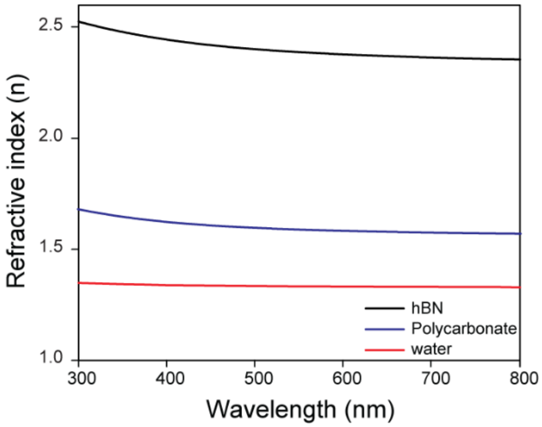

Supplementary Figure S13. Optical constants of hBN, polycarbonate, and water used for simulation.

Supplementary Note 1

Total energy (E_{tot}) minimization of bubble

E_{tot} can be described by four energy terms when no external strain is applied: (i) in-plane elastic energy (E_{el}), (ii) bending energy (E_{bend}), (iii) adhesion energy between the bent multilayer and the substrate (E_{adh}), and (iv) free energy of material inside the bubble ($E_b(V)$):

$$E_{tot} = E_{el} + E_{bend} + E_{adh} + E_b(V) = c_1' Y \frac{h^4}{r^2} + c_2' \kappa \frac{h^2}{r^2} + c_3 \gamma r^2 + E_b(V) \qquad (SI1)$$

$$P = -\frac{\partial E_b(V)}{\partial V}, V = c_4 h r^2 \quad (SI2)$$

Here, r and h are radius and height of the bubble, Y is the in-plane stiffness, κ the bending rigidity, γ the adhesion energy, V the volume of the bubble, P the pressure inside the bubble, and c_1, c_2, c_3, c_4 are constants(*2*). As κ originates from vdW interlayer interaction, no additional term of vdW interaction is necessary. At equilibrium, E_{tot} is minimized with respect to r and h (*2*, *7*, *8*).

$$\frac{\partial E_{tot}}{\partial h} = 4c_1' Y \frac{h^3}{r^2} + 2c_2' \kappa \frac{h}{r^2} - c_4 r^2 P = 0 \qquad (SI3)$$

$$\frac{\partial E_{tot}}{\partial r} = -2c_1' Y \frac{h^4}{r^3} - 2c_2' \kappa \frac{h^2}{r^3} + 2c_3 \gamma r - 2c_4 h r P = 0 \qquad (SI4)$$

From this, we can write,

$$2\frac{\partial E_{tot}}{\partial h} \times h - \frac{\partial E_{tot}}{\partial r} \times r = 10c_1' Y \frac{h^4}{r^2} + 6c_2' \kappa \frac{h^2}{r^2} - 2c_3 \gamma r^2 = 0 \quad (SI5)$$

By defining the aspect ratio $a = \frac{h}{r}$, equation (SI5) can be rewritten as:

$$10\mathrm{c}_1'Yh^2a^2 + 6\mathrm{c}_2'\kappa a^2 - \frac{2\mathrm{c}_3\gamma h^2}{a^2} = 0 \quad (SI6)$$

$$a^4 = \frac{\mathrm{c}_3\gamma h^2}{5\mathrm{c}_1'Yh^2 + 3\mathrm{c}_2'\kappa} = \frac{\gamma h^2}{\mathrm{c}_1 Yh^2 + \mathrm{c}_2\kappa} \quad (SI7)$$

As shown in Supplementary Table 1, for monolayer, parameters of Y_{mono}, c_1, γ are used, and for multilayer, parameters of $Y_{multi}, \mathrm{c}_1, \gamma, \mathrm{c}_2, \kappa$ are used.

In addition, the vdW pressure is calculated using equation SI3 or SI4.

$$P = \left(\frac{4\mathrm{c}_1 Y}{\mathrm{c}_4 h} + \frac{2\mathrm{c}_2\kappa}{\mathrm{c}_4 h^3}\right)\left(\frac{h}{r}\right)^4 \quad (SI8)$$

Therefore, the pressure of the bubble in Figure 4a of the main text before and after irradiation can be calculated as h and r of each state are measured. Before irradiation, the dimensions of the bubble are measured as $r_1 = 0.9\ \mu\mathrm{m}, h_1 = 0.02\ \mu\mathrm{m}$, which gives $V_1 = 0.0257\ \mu\mathrm{m}^3$ and $P_1 = 5.04\ \mathrm{MPa}$. After irradiation, the bubble expands to $r_2 = 1.4\ \mu\mathrm{m}, h_2 = 0.05\ \mu\mathrm{m}$, which gives $V_2 = 0.150\ \mu\mathrm{m}^3$ and $P_2 = 2.11\ \mathrm{MPa}$. Under the assumption that gas molecules fill the increased volume ($\Delta V = 0.124\ \mu\mathrm{m}^3$) at room temperature (during AFM measurement), 6×10^7 molecules would be trapped in its increased volume of $0.124\ \mu\mathrm{m}^3$ of under 2.11 MPa using the ideal gas law $\Delta(PV) = nRT$.

Supplementary Note 2

Beam-induced bubble expansion

To uncover the underlying mechanism of the expansion, we vary the electron beam accelerating voltage (1 – 10 keV) in SEM and find that the expansion requires an accelerating voltage high enough for the electron beam to penetrate the thickness (t) of the top multilayer, reaching the material inside the bubble. This is more clearly observed in bubbles in a WS_2/WS_2 double-multilayer due to their high atomic number, as the expansion of $t = 60$ nm bubbles is not observed under 1-2 kV electron beam with a calculated penetration depth <50 nm. As the energy of the beam increases, reaching the interface between two multilayers, the bubbles in WS_2/WS_2 double-multilayer then start to expand. The expansion speed and change in volume of the bubbles depends on the current density (100 – 500 pA) and the dwell time (25 ms/pixel) at 3 – 10 kV, where electron flux is $10^4 - 10^5$ electrons/nm^2. These results imply that interaction of the beam with the material trapped inside the bubble is critical for expansion. Bubble expansion is also observed in TEM, but with smaller (below 10x less) expansion speed and change in volume of the bubble. We presume that this is due to the small interaction volume at high kV as elaborated in Supplementary Figure 7.

	In-plane stiffness	c_1	Adhesion energy	Bending rigidity
Monolayer	$Y_{mono} = 22(\pm 6)\text{eV}/\text{Å}^2$ [26, 39]	1.55 [26]	$\gamma = 0.005\ \text{eV}/\text{Å}^2$ [26]	-
Multilayer	$Y_{multi} = Y_{mono}t$ (t: thickness)	1.55 [26]	$\gamma = 0.005\ \text{eV}/\text{Å}^2$ [26]	κ

Supplementary Table 1. Parameters used to calculate aspect ratio in the main text. First, three parameters c_1, Y_{mono}, and γ are adopted from Ref. 26 for the case of a monolayer hBN. Then, to expand the model to the case of multilayers, new terms Y_{multi} and κ are used which account for the different mechanical properties between monolayer and multilayer. Adhesion energy (γ)

should be independent of thickness as it only depends on the interface surface area. c_2 is a dimensionless coefficient that depends on out-of-plane displacement. Unlike c_1, which depends on in-plane displacement, c_2 largely depends on the deflection profile of the bubble. Therefore, c_2 varies with thickness and radius of bubbles as we found in Figure 4c-d.

References

1. L. Museur, E. Feldbach, A. Kanaev, Defect-related photoluminescence of hexagonal boron nitride. *Phys. Rev. B*. **78**, 155204 (2008).
2. E. Khestanova, F. Guinea, L. Fumagalli, A. K. Geim, I. V Grigorieva, Universal shape and pressure inside bubbles appearing in van der Waals heterostructures. *Nat. Commun.* **7**, 1–10 (2016).
3. G. Wang, Z. Dai, J. Xiao, S. Feng, C. Weng, L. Liu, Z. Xu, R. Huang, Z. Zhang, Bending of multilayer van der Waals materials. *Phys. Rev. Lett.* **123**, 116101 (2019).
4. D. Drouin, A. R. Couture, D. Joly, X. Tastet, V. Aimez, R. Gauvin, CASINO V2. 42—a fast and easy-to-use modeling tool for scanning electron microscopy and microanalysis users. *Scanning J. Scanning Microsc.* **29**, 92–101 (2007).
5. N. Sultanova, S. Kasarova, I. Nikolov, Dispersion proper ties of optical polymers. *Acta Phys. Pol. A Gen. Phys.* **116**, 585 (2009).
6. G. M. Hale, M. R. Querry, Optical constants of water in the 200-nm to 200-μm wavelength region. *Appl. Opt.* **12**, 555–563 (1973).
7. K. Yue, W. Gao, R. Huang, K. M. Liechti, Analytical methods for the mechanics of graphene bubbles. *J. Appl. Phys.* **112**, 83512 (2012).
8. P. Wang, W. Gao, Z. Cao, K. M. Liechti, R. Huang, Numerical analysis of circular graphene bubbles. *J. Appl. Mech.* **80** (2013).
